# Evaluation of integrated respiratory gating systems on a Novalis Tx system

**DOI:** 10.1120/jacmp.v12i3.3495

**Published:** 2011-04-04

**Authors:** Zheng Chang, TongHai Liu, Jing Cai, Qing Chen, Zhiheng Wang, Fang‐Fang Yin

**Affiliations:** ^1^ Department of Radiation Oncology Duke University Durham North Carolina 27710 USA; ^2^ Department of Radiation Oncology Shandong Cancer Hospital and Institute Jinan Shandong 250117 China

**Keywords:** respiratory gating system, ExacTrac, RPM, Novalis Tx system

## Abstract

The purpose of this study was to investigate the accuracy of motion tracking and radiation delivery control of integrated gating systems on a Novalis Tx system. The study was performed on a Novalis Tx system, which is equipped with Varian Real‐time Position Management (RPM) system, and BrainLAB ExacTrac gating systems. In this study, the two systems were assessed on accuracy of both motion tracking and radiation delivery control. To evaluate motion tracking, two artificial motion profiles and five patients' respiratory profiles were used. The motion trajectories acquired by the two gating systems were compared against the references. To assess radiation delivery control, time delays were measured using a single‐exposure method. More specifically, radiation is delivered with a 4 mm diameter cone within the phase range of 10%–45% for the BrainLAB ExacTrac system, and within the phase range of 0%–25% for the Varian RPM system during expiration, each for three times. Radiochromic films were used to record the radiation exposures and to calculate the time delays. In the work, the discrepancies were quantified using the parameters of mean and standard deviation (SD). Pearson's product‐moment correlational analysis was used to test correlation of the data, which is quantified using a parameter of *r*. The trajectory profiles acquired by the gating systems show good agreement with those reference profiles. A quantitative analysis shows that the average mean discrepancies between BrainLAB ExacTrac system and known references are 1.5 mm and 1.9 mm for artificial and patient profiles, with the maximum motion amplitude of 28.0 mm. As for the Varian RPM system, the corresponding average mean discrepancies are 1.1 mm and 1.7 mm for artificial and patient profiles. With the proposed single‐exposure method, the time delays are found to be 0.20±0.03 seconds and 0.09±0.01 seconds for BrainLAB ExacTrac and Varian RPM systems, respectively. The results indicate the systems can track motion and control radiation delivery with reasonable accuracy. The proposed single‐exposure method has been demonstrated to be feasible in measuring time delay efficiently.

PACS numbers: 87.56.bd, 87.56.‐v, 87.55.‐x

## I. INTRODUCTION

Respiratory motion is a major source of target uncertainty of external beam treatments of thoracic and abdominal tumors.^(1)^ The uncertainty caused by respiration needs to be minimized in order to deliver radiation precisely to the tumor while sparing adjacent healthy tissue.^(2)^ In the past few years, various techniques have been proposed to overcome this challenge, which include but are not limited to respiratory gating, breath‐hold, forced shallow breathing, and respiration synchronized techniques.^(^ ^3^ ^–^ ^7^ ^)^ Among these techniques, respiratory gating is commonly used, in which radiation is delivered within a particular portion of the patient's breathing cycle or a duty cycle.^(^ ^6^ ^–^ ^12^ ^)^ The gating method usually obtains a respiratory motion signal from an external surrogate. For example, patient abdominal movement due to respiration can be monitored by a video camera with an infrared marker on the patient's abdomen.^(^ ^6^ ^–^ ^9^ ^)^ Since a gating system plays a significant in role in treatment delivery, it is essential to evaluate and verify the accuracy of the gating system before clinical use.

Among the characteristics of a gating system, motion tracking and radiation delivery control are two fundamental features. In a respiratory gated radiotherapy, the gating phase for treatment delivery is required to coincide with the corresponding phase as determined during simulation. In this regard, time delay is the most basic parameter controlling delivery accuracy, which must be measured and verified. In this work, an efficient single‐exposure method is used to measure the time delay. In addition to radiation delivery control, motion tracking is another fundamental feature of a gating system. Although qualitative studies of motion tracking have been demonstrated in literature,^(3)^ few quantitative analyses are presented. In this work, integrated gating systems on the Novalis Tx system were evaluated using both quantitative motion tracking analyses and efficient time delay measurements.

## II. MATERIALS AND METHODS

Evaluation of the integrated respiratory gating system was performed on a Novalis Tx system, which is equipped with Varian real‐time position management (RPM) gating system (Varian Medical Systems, Palo Alto, CA) and BrainLAB ExacTrac gating system (BrainLAB, Heimstetten, Germany).

### A. Varian RPM respiratory gating system

Varian RPM respiratory gating system consists of a marker block, an infrared (IR) light ring that emits IR light, a charge‐coupled detector (CCD) as a tracking camera used to visualize the relative position of the block, and a workstation that displays and records the motion data. The marker block is often placed on the patient's chest or abdomen. The reflective fiducial markers are tracked using the IR light source and CCD detector. In this way, the motion of the block is considered as a surrogate for respiratory‐induced tumor motion.

### B. BrainLAB ExacTrac gating system

Similarly, BrainLAB ExacTrac system includes IR and video detectors, as well as a set of radiographic kV devices. The radiographic kV devices consist of two floor‐mounted diagnostic kV X‐ray tubes and two corresponding ceiling‐mounted amorphous silicon X‐ray imagers. Matching tools are available to align kV images with corresponding DRRs. The IR cameras are used to monitor IR reflective body markers placed on the patient as a surrogate for tumor motion. A nice feature of the BrainLAB ExacTrac system is the ability to correlate internal tumor motion with external IR markers using X‐ray imaging. From this perspective, the BrainLAB ExacTrac system is one step improved over the RPM system regarding real‐time tumor tracking.

### C. Method for efficient time delay measurements

**Figure 1:**
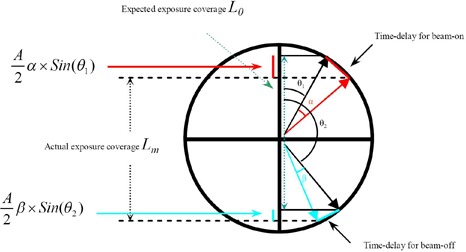
Illustration of the principle of the time delay measurement method when a sinusoidal profile is used as motion trajectory.

To assess radiation delivery control, a single‐exposure method is proposed to measure time delays for both gating systems. The principle of the proposed method is illustrated in Fig. 1, when a sinusoidal profile is used as the motion trajectory. As shown in Fig. 1, the arrows in black indicate the temporal points for beam‐on and beam‐off in theory. However, due to the existence of time delay, the temporal point for beam‐on is postponed by a short period of time for beam‐on, as indicated by the arrow in red. Similarly, the temporal point for beam‐off is extended by another short period of time for beam‐off, as indicated in blue. In this case, the beam‐on time delay can be characterized by an angular range of α, and the beam‐off time delay can be quantified by an angular range of β. Since a sine function features a constant angular speed at a fixed period, the expected exposure coverage can be described by the following equation, assuming time delays much less than the period:

(1) $$\frac{A}{2}\alpha \times Sin(\theta_{1})+L_{m}-\frac{A}{2}\beta \times Sin(\theta_{2})=L_{0}$$

where *A* stands for the peak‐to‐peak amplitude of the sine profile, $L_{m}$ is the actual exposure length (which can be measured using a recording device such as radiochromic films or electronic portal imager), $L_{0}$ is the expected exposure length from beam‐on phase indicated by the angle of $\theta_{1}$ to the beam‐off phase indicated by the angle of $\theta_{2}$. Given a known period of the sine profile, the time delays for beam‐on and beam‐off can be expressed as $\frac{\alpha T}{2\pi }\text{and}\frac{\beta 2}{2\pi }$, respectively.

Based on the above equation, two unknown variables α and β can simply be determined using two equations through two exposure measurements with two different phase ranges. Assuming time delays for beam‐on and beam‐off are identical, the two unknown variables α and β can be turned into one unknown variable. In this way, the time delay for radiation treatment delivery can be calculated using a single exposure, provided that the temporal points for beam‐on and beam‐off are not symmetric. In this work, the single‐exposure method is proposed to determine time delays for both BrainLAB ExacTrac and Varian RPM gating systems, assuming the time delays for beam‐on and beam‐off are equal and independent of selection of a phase range.

### D. Phantom experiments for motion tracking assessment

**Figure 2:**
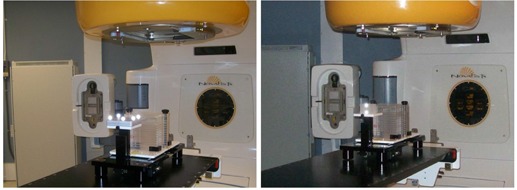
Motion phantom platform setups used for the phantom measurements of BrainLAB ExacTrac gating system (left) and Varian RPM gating system (right).

To evaluate motion tracking, two artificial motion profiles and five patients' respiratory profiles were used, which were realized on a motion phantom platform (BrainLAB, Heimstetten, Germany) as shown in Fig. 2. More specifically, the two artificial profiles are generated from two mathematical modes: sine and (1‐cosine)^2^, each with a period of 5 sec and amplitude of 28.0 mm. The five patient profiles were acquired previously, each with the maximum amplitude of 28.0 mm. The tracking trajectories of the gating systems were compared against the references, and the discrepancies were quantitatively analyzed.

### E. Phantom experiments for time delay measurements

As described previously, the single‐exposure method is proposed in this work to measure the time delays for both BrainLAB ExacTrac and Varian RPM systems. In this work, radiation was delivered in two different gated manners with a 4 mm diameter cone, which was triggered by a sine profile: the first was delivered by the BrainLAB ExacTrac system within the phase range of 10%–45%, while the second was delivered by the Varian RPM system within the phase range 0%–25%, both during expiration. Radiochromic films were used in this work to record the radiation exposures, which were measured to calculate the time delays. More specifically, for each exposure measurement, one external beam therapy (EBT) radiochromic film with a dimension of 10 cm×20 cm was sandwiched with two plastic plates and placed on the top of the motion phantom as shown in Fig. 2. A radiation beam of monitor units (MU) of 1000 was then delivered with a 4 mm diameter cone in the gated manner either by BrainLAB ExacTrac or Varian RPM systems, as described above. After the exposure, the film was scanned with a VXR‐16 DosimetryPro film digitizer (VIDAR, Herndon, VA). Due to the introduced sinusoidal motion during the radiation delivery, the exposure in the film appears as a long, narrow band with both round ends. To quantify the coverage of the exposure, the central profile of the long band was plotted, and the local maximal intensity (excluding background) of each end was indentified. The distance between the points of the half local maximal intensity of both ends was measured as the coverage of the exposure. The above procedure was repeated three times for both BrainLAB ExacTrac and Varian RPM systems. The use of a cone with a known diameter served the purpose of calibration during film analysis.

### F. Statistical Analysis

In this work, the discrepancies were quantified using mean value and standard deviation (SD). Linear regression analyses of the profiles measured by using the gating systems as a function of the known reference profiles were performed. Pearson's product‐moment correlational analysis was used to test correlation of the data, which is quantified using a parameter of *r*.

## III. RESULTS

**Figure 3:**
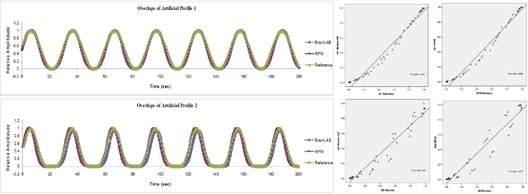
Overlays of trajectory profiles and the corresponding linear regression analyses: comparison among BrainLAB ExacTrac, Varian RPM, and known reference for two artificial motion profiles.

Figure 3 shows overlays of trajectory profiles measured by using the two gating systems and the reference profiles for two artificial motion profiles. Each overlay is accompanied by a corresponding linear regression analysis which correlates the measurements with the known references. As illustrated in the figure, the trajectory profiles acquired by the gating systems show good agreement with those reference profiles. A quantitative analysis shows that the average mean discrepancy of the trajectory profiles between the BrainLAB ExacTrac gating system and the reference profiles is 1.5 mm. The corresponding the parameter *r* ranges from 0.975 to 0.989. Similarly, the average mean discrepancy of the trajectory profiles between Varian RPM gating system and the reference profiles is 1.1 mm, which is about 4.1% of the motion amplitude. The corresponding parameter *r* ranges from 0.968 to 0.990. All the results were summarized in Table 1 for easy comparison.

**Table 1:** Tracking discrepancies of Exac Trac and RPM systems with references for two artificial profiles.

	*Comparison Between Profiles Tracked by BrainLAB ExacTrac and References* (amplitude=28.0 *mm)*		*Comparison Between Profiles Tracked by Varian RPM and References* (amplitude=28.0 *mm)*	
*Motions*	*Difference in mm (mean, SD)*	*r‐value*	*Difference in mm (mean, SD)*	*r‐value*
Artificial Motion 1 (sine)	1.0, 1.6	0.989	0.9,1.6	0.990
Artificial Motion 2				
${\left(1-\text{cosine}\right)}^{2}$	2.0, 2.3	0.975	1.4, 2.7	0.968
Average	1.5, 2.7	N/A	1.1, 2.1	N/A

**Figure 4:**
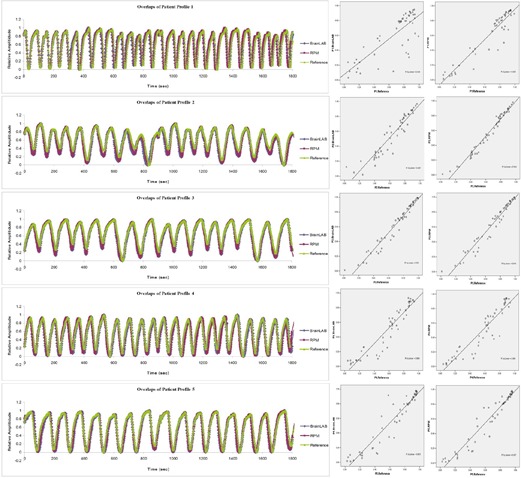
Overlays of trajectory profiles and the corresponding linear regression analyses: comparison among BrainLAB ExacTrac, Varian RPM, and known reference for five patient motion profiles.

Figure 4 shows overlays of trajectory profiles measured by the two gating systems and the reference profiles for five patient motion profiles, along with the corresponding linear regression analyses. As illustrated in Table 2, the average mean discrepancy of the trajectory profiles between BrainLAB ExacTrac respiratory gating system and the reference profiles is 1.9 mm. The corresponding the parameter *r* ranges from 0.800 to 0.975. In contrast, the average mean discrepancy of the trajectory profiles between Varian RPM gating system and the reference profiles is 1.7 mm. The corresponding parameter *r* ranges from 0.932 to 0.982. The trajectory profiles acquired by the two gating systems have demonstrated to be consistent with those known references.

**Table 2:** Tracking discrepancies of Exac Trac and RPM systems with references for five patient profiles.

	*Comparison Between Profiles Tracked by BrainLAB ExacTrac and References (maximum* amplitude=28.0 *mm)*		*Comparison Between Profiles Tracked by Varian RPM and References (maximum* amplitude=28.0 *mm)*	
*Patients*	*Difference in mm (mean, SD)*	*r‐value*	*Difference in mm (mean, SD)*	*r‐value*
Patient 1	1.6, 5.2	0.800	1.5, 2.9	0.946
Patient 2	2.2, 2.7	0.942	2.0, 1.8	0.982
Patient 3	1.9, 2.1	0.975	1.6, 2.2	0.973
Patient 4	2.2, 2.0	0.943	1.9, 3.3	0.932
Patient 5	1.7, 3.2	0.945	1.4, 2.7	0.963
Average	1.9, 3.2	N/A	1.7, 2.6	N/A

**Figure 5:**
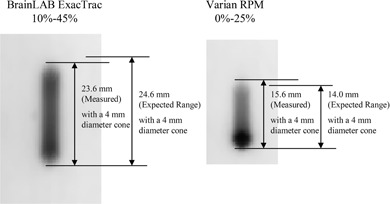
Radiation exposures measured by radiochromic films for gated deliveries by BrainLAB ExacTrac system in the duty cycle from 10%–45% phase range (left) and Varian RPM system in the duty cycle from 0%–20% phase range (right), for the artificial sine motion profile with the period of 5 sec and the amplitude of 28.0 mm.

Figure 5 shows two radiation exposures measured with radiochromic films: one delivered by BrainLAB ExacTrac gating system within the phase range of 10%–45%, and the other delivered by Varian RPM gating system within the phase range of 0%–25%, both during expiration. Exposure coverage is measured to be 23.6±0.1 mm for BrainLAB ExacTrac and 15.6±0.1 mm for Varian RPM systems. With the proposed single‐exposure method, the time delays are found to be 0.20±0.03 sec and 0.09±0.01 sec for BrainLAB ExacTrac and Varian RPM systems, respectively.

## IV. DISCUSSION

In this work, the trajectory profiles acquired by the gating systems show good agreement with those reference profiles. Quantitative analyses show that the average mean discrepancies between the gating systems and known references are less than 1.5 mm and 2.0 mm for artificial and patient profiles, with the maximum motion amplitude of 28.0 mm. It should be noted that the discrepancies include those caused by the motion phantom platform itself and, as such, the actual discrepancies of the gating systems might be less than those reported in this work. We think that the value of the quantitative motion tracking analyses is to demonstrate the possible maximal error of which clinicians should be aware in clinical practice. In addition to motion tracking, radiation delivery control was evaluated by measuring the parameter of time delay with the proposed single‐exposure method. In this work, the time delays are found to be less than 0.20 sec for both gating systems: 0.20±0.03 sec for BrainLAB ExacTrac gating system and 0.09±0.01 sec for Varian RPM gating system, respectively. This finding is generally consistent with data reported by other works published in the literature.^(^ ^4^ ^–^ ^7^ ^)^ Specifically, Jin and Yin^(4)^ reported that the time delay of a linear accelerator (LINAC)‐based BrainLAB gating system was 0.17±0.03 sec; Smith and Becker^(5)^ reported that the time delay of a LINAC‐based Varian RPM gating system varied from 0.07 to 0.10 sec. Although the methodology and systems used in the previous studies are different from those in this work, the results obtained using the proposed single‐exposure method are generally consistent with those reported by other investigators.

The single‐exposure method is proposed to determine the time delays for gating system based on the assumption that the time delays are much less than the period used. The assumption generally holds true when a period comparable to that of normal breathing cycle is chosen. In the rare occasion where a time delay is large, a much longer period (e.g., 20 sec) can be selected so that the above assumption can still be valid. Although a period of 5 sec is demonstrated in this work, an arbitrary long period can always be used to improve the accuracy of the calculation with an adequate measuring device.

In this work, time delays for beam‐on and beam‐off are assumed to be identical, and a single‐exposure method is used to determine time delays. If the time delays for beam‐on and beam‐off are to be determined separately, they can be calculated using two exposures with two different phase ranges. When multiple exposures are available, the time delays can always be determined by using least‐square minimization with improved accuracy. Although a sinusoidal profile is used in the study, any other known profiles can be used as long as the time delays can be correlated with the corresponding spatial discrepancies.

In the phantom experiments for time delay measurements, radiation was delivered in a gated manner with a 4 mm diameter cone, and EBT radiochromic films were used to record the radiation exposures. The exposed films were then scanned with a VIDAR film digitizer. Since the coverage of the exposure was measured based on intensity as described previously, the accuracy of the measurements depends on how accurate half local maximal intensity of both ends of the exposure band can be determined. In this study, a 4 mm cone was used, which is the smallest cone available in our clinic, and it provides a sharp penumbra of about 1.5 mm — the distance between 80% and 20% of the maximal intensity of the 4 mm cone profile. This implies that 5% discrepancy in intensity corresponds to around 0.1 mm uncertainly in the spatial measurement. A discrepancy of 5% of the maximal intensity is rather large and can be detectable, so the film measurement used herein can be considered adequate for the exposure coverage quantification. It should be noted that the same spatial discrepancy in the exposure coverage measurement may lead to different discrepancies in time delay depending on the phase range selected, which can also be demonstrated by the non‐linearity of Eq. (1). For example, in this work, the spatial discrepancy of about 0.1 mm yielded different temporal discrepancies in time delay: 0.03 sec for the phase range of 10%–45% and 0.01 sec for the phase range of 0%–25%. The selection of a proper phase range should, therefore, be taken in account when it comes to a time delay measurement.

In clinical practice, clinical target volume (CTV) or internal target volume (ITV) often needs an expansion to account for setup uncertainties (setup margin) to generate a new planning target volume (PTV) for use in the treatment planning.^(^ ^13^ ^–^ ^14^ ^)^ This expansion ensures that CTV/ITV can be covered by the prescribed dose during treatments. When a gating treatment is selected for a patient, the expansion from CTV/ITV to PTV will include the uncertainties introduced by the gating system, in additional to the standard setup margin. Various methods have been introduced to account for overall spatial uncertainties.^(15)^ Given the uncertainties that cannot be ignored caused by motion tracking and time delay as reported in the work, the margin from CTV/ITV to PTV should be larger than standard setup margin when a gating treatment is used.

Since respiratory gating has been adopted in clinical practice for various advanced radiotherapy techniques (such as hypofractionated stereotactic body radiotherapy), it is clinically crucial to verify time delay for any clinical gating system. The efficient feature of the single‐exposure method would provide clinical users with a convenient and efficient option, especially in a busy clinic.

## V. CONCLUSIONS

In this work, accuracy of motion tracking and radiation delivery control in the BrainLAB ExacTrac and Varian RPM respiratory gating systems was investigated on a Novalis Tx system. The results indicate the systems can track motion and control radiation delivery with reasonable accuracy. The proposed single‐exposure method has been demonstrated to be feasible in measuring time delay in an efficient manner.
